# Correlation of ultrasound placental diameter & thickness with gestational age

**DOI:** 10.12669/pjms.36.5.1938

**Published:** 2020

**Authors:** Ngozi R. Njeze, Joseph O. Ogbochukwu, Josephat M. Chinawa

**Affiliations:** 1 Ngozi R. Njeze, Senior lecturer Department of Radiation Medicine, University of Nigeria Medical School, Nsukka, Nigeria; 2 Joseph O. Ogbochukwu, Consultant Radiologist, Department of Radiology, Federal Medical Center Nguru, Nigeria; 3 Josephat M. Chinawa Associate Professor, Department of Pediatrics, University of Nigeria Medical School, Nsukka, Nigeria

**Keywords:** Placenta, Ultrasonography, Gestational age, Enugu-Nigeria

## Abstract

**Background & Objectives:**

Estimation of fetal maturity is common in obstetric practice especially when the women do not keep accurate menstrual records. An accurate establishment of expected date of delivery is fundamental to the management of both high risk and normal pregnancies. The objective of this study was to determine the placental diameter (PD), placental thickness (PT) and to establish a correlation between PD, PT and gestational age.

**Methods:**

This is an observational cross-sectional study that examines by means of ultrasonography the correlation between placental diameter and thickness with gestational age in Enugu, South East, Nigeria.

**Results:**

A total of 400 healthy subjects were recruited in 3^rd^ trimester of pregnancy having fulfilled the inclusion criteria. PD and PT in this study did not correlate with parity. There is a linear increase of gestational age and placental thickness and diameter. These increases heighten between 38^th^ week gestation and 40 weeks’ gestation. 205.0±1.4, 43.00±0.0 to 215.0±1.4, 46.00±2.8 respectively.

**Conclusion:**

Placental thickness and Placental diameter can be used to predict gestational age. It is therefore advised to use PT & PD in ultrasound obstetric assessment especially when Last menstrual period (LMP) is not clear.

## INTRODUCTION

The gestational age is necessary in the management of pregnancy. It is usually estimated by ultrasound using parameters like femur length, bi-parietal diameter, abdominal circumference which have their limitation. It is imperative to seek other parameters to compliment them in predicting gestational age.^1^

The placenta is highly vascular and ensures adequate interaction between the mother and fetus.^1^-^5^ It is known that a healthy and normal placenta enhances fetal growth and is key in good perinatal result.^2^ Fetal growth is affected when placental function is not optimal. As a result, alteration in placental measurement is an indicator of abnormal fetal growth.^2^-^9^ Placental thickness is a key factor in perinatal outcome since it affects fetal development. At birth, placental thickness is about 3cm while diameter is 15 - 25cm.^10^ Ultrasound done at 36wks measuring 18cm in diameter and 2cm at 36wks predicts a low birth weight neonate.^11^ Small sized placentas may result in intra-uterine growth restriction, chromosomal anomalies, severe maternal diabetes mellitus, preeclampsia, chronic intra uterine fetal infections, polyhydramnios.^11^

Large sized placentas (more than 4cm thickness) at term are mostly due to diabetes mellitus, perinatal infections, & hydrops fetalis.^11^,^12^ The assessment of PD and PT will enable the obstetrician achieve successful fetal outcome. Important clinical decisions like gestational age at which caesarian section, elective induction of labor depend on accurate timing of gestational age.^12^

There is a yearning gap created by other methods of assessment of gestational age. For instance, the use of last menstrual period (LMP), symphysio-pubic fundal height (SFH) and Ballard Score (BS) at delivery, though often used they have limited role and lack of precision in estimating gestational age is gross.^13^ A well-coordinated ultrasound facility with its attendant skills will indeed be very essential in the study and management of gestational age in newborns.^13^

Jehan et al.^14^ in their study, compared the accuracy of the last menstrual period (LMP) and symphysis-fundal height (SFH) in the estimation of gestational age (GA), using ultrasound (US) scan as reference. They concluded that though SFH was a more accurate method of assessing gestational age than LMP, however, neither of the above tools were as accurate as the use of ultrasonography.

This study therefore aimed to find the correlation if any between placental diameter, thickness with gestational age and match placental health with placental diameter and thickness to gestational age and fetal outcome. It also hopes to guide obstetricians practicing in peripheral health centers to alert the neonatologists of those fetuses that may require extra care at birth.

## METHODS

This is an observational cross-sectional study that examines by means of ultrasonography the correlation between placental diameter and thickness with gestational age in the University of Nigeria Teaching Hospital, Enugu, South East, Nigeria.

### Study Area

The study was carried out among pregnant mothers who attend ante-natal clinic in UNTH Enugu, Nigeria.

This study comprised four hundred women with viable singleton pregnancy who met the inclusion criteria. They were consecutively recruited over one-year period.

This is an observational cross sectional study which spanned over one year period and involved 400 women with singleton pregnancies in their 3^rd^ trimester. They were scanned at They were scanned at 32 weeks’ gestation, 36 weeks, and other times within the third trimester.

The gestational age is frequently over or under estimated as many women do not know their last menstrual period. Irregular menstruation is also going to add to this difficulty in accessing GA.

### Measurement of placental diameter & thickness

A mobile ultrasound machine was used to acquire data; SONOSITE M-Turbo (made in USA). Curvilinear probe frequency of 3.5 – 5mHz with the participant lying in supine position on the examination couch, coupling gel was applied on the abdomen after exposing it. Placental thickness & diameter were measured & recorded in the data sheet.

It was done parallel to the length of the chorionic surface from upper to lower limit of the placenta. The placenta was measured by split screen method whereby the upper limit to midline was sited in one part of the screen; the other half of the screen was used to measure from the midline to the lower limit of the screen.

The placental thickness was measured perpendicularly at the level of the umbilical cord insertion from feto-placental surface to placenta-endometrial surface. Inclusion criteria were normal viable singleton pregnancy, at 28-41 weeks. Women who were not sure of their dates were not selected same as those with uterine masses or mothers with diabetes mellitus or sickle cell disease.

### Data analysis

All data were analyzed using the Statistical Package for Social Sciences program (SPSS version 20 Chicago.) Chi-square was used to test significant association for qualitative variables while multivariate logistic regression was used to determine correlates. A p-value less than 0.05 was accepted as significant for each statistical test.

### Ethical Considerations

Ethical clearance was dully obtained from the ethical and research committee of the University of Nigeria Enugu with IRB number of IRB00002323 and issue date of 17th May 2019. Participants were tutored about the study and their confidentiality assured.

## RESULTS

A total of 400 healthy subjects were recruited in 3^rd^ trimester of pregnancy having fulfilled the inclusion criteria. Table-I shows demographic data of pregnant females aged between 20–44 years with a mean of 29.28 year ± 3.52. Modal age group was 30-34 years, 161(40.3%) followed by 25–29 years, age group which was 144 (36.0%) women. The least number was the 40-44 age group which had 3 subject (0.7%).

**Table-I:** Demographic characteristics of the study population.

Variables	Frequency	Percentages (%)
***Age***		
20 -24	50	12.5
25-29	144	36
30-34	161	40.3
35-39	42	10.5
40-49	3	0.7
Total	400	100
***Body mass index***		
Normal	151	37.7
Overweight	165	41.3
Obesity	84	21.0
Total	400	100

The number of subjects decreased with parity. Parity ranged from one to five. Multiparous women were 166 (41.5%). Nulliparous women were 134 (35.5%). 98 primi-parous women were 24.5% of the study population. There were two grand multiparous women (0.5%).

**Table-II:** Obstetrics characteristics of the study population.

Variables	Frequency (n=400)	Percentages(%)
***Parity***		
0 (nulli-parous)	134	33.5
1 (primi-parous)	98	24.5
2 (multi-parous)	80	20.0
3 (multi-parous)	55	13.8
4 (multi-parous)	31	7.7
5 (grand-multiparous)	2	0.5
Total	400	100
***Mode of delivery***		
(spontaneous vaginal delivery	344	86.0
Caesarian section	56	14.0
***Gestational age groups (weeks)***		
28-31	107	26.7
32-35	147	36.7
36-39	129	32.3
40-41	17	1.3
***Gestation at delivery (weeks)***		
31-35	58	14.5
36-40	274	68.5
40-41	68	17.0
***Birth weight (kg)***		
< 2.5	37	9.3
>2.5	363	90.7
***Post-delivery gender***		
Male	210	52.5
Female	190	47.5

PD & PT in this study did not correlate with parity. There is a linear increase of gestational age and placental thickness and diameter. These increases heighten between 38^th^ week gestation and 40 weeks’ gestation. 205.0±1.4, 43.00±0.0 to 215.0±1.4, 46.00±2.8 respectively.

**Table-III:** Mean placental & foetal parameters by GA.

EGA (wks)	No. of subjects (N)	Mean PD (mm)±SD	Mean PT (mm)±SD
28	25	167.8±25.8	32.3±1.2
29	23	169.0±9.4	32.75±2.6
30	24	180.0±3.0	34.00±1.1
31	35	183.0±1.8	35.00±1.3
32	50	184.7±2.3	35.88±1.5
33	30	188.6±4.0	37.00±3.3
34	34	192.0±15.3	39.06±4.7
35	33	195.0±8.8	40.50±3.4
36	45	194.2±7.8	40.00±1.2
37	35	193.7±4.8	39.00±0.7
38	31	205.0±1.4	43.00±0.0
39	18	215.0±1.4	46.00±2.8
40	12	208.0±2.8	44.50±0.7
41	5	207.0±2.3	43.50±0.4

Total	400	186.0±9.1	36.5±2.9

## DISCUSSION

Placental thickness is known to increase progressively with gestational age and at term the placenta is known to attain a weight of 500g.^15^,^16^ Its thickness tends to increase steadily with GA in a linear fashion. This progressive increase is by about one millimeter per week. The gestational age (in weeks) is same as placental thickness ± 10 mm. Anteriorly located placenta is usually thinner than posterior placenta by about 0.7cm.^17^

Use of fetal parameters though helpful will need PT and PD to enhance accuracy of gestational age. Thurston^18^ and Anna^19^ variously reported that placental thickness indeed parallels gestational age and that it has a very high correlation with GA.^18^ We also noted a strong correlation and linear increase of PT and PD with GA. Khanal^20^ and colleagues were hopeful that placental thickness would be used to estimate gestational age.

Mathai^21^ also reported that PT increases with gestational age.^21^ Placental thickness of >40mm at term is associated with gestational diabetes, intrauterine infection and hydrops fetalis. We obtained higher figures in our work similar to Karthikeyan.^22^ This supports the impression that placental thickness is most likely higher in negroes. Uterine contractions may alter placental thickness giving a false increase in thickness same as severe polyhydramnios and may decrease PT. Oligohydramnios may give a false increase in size. Kullman et al.^23^ showed that placental thickness less than 25mm in 3^rd^ trimester is associated with intrauterine growth retardation. None of our subjects had either polyhydramnios or oligohydroaminios. Mital^24^ in India and Kadam^1^ also in India concluded that placental thickness is important in estimating gestational age especially when patients are not sure of their dates.

We noted from our study that parity does not correlate with PD and PT. There is an increase of placental thickness especially at the second and third trimester. These findings are also in keeping with ours. However, it is expedient to note that increased placental thickness is not diagnostic of any specific disorder but may contribute to the management of a fetus at risk. In addition, increases in placental thickness during second trimester is due to over-inflation of the intervillous space by maternal blood rather than by adaptive formation of functional placental tissue. Higgins^25^ and colleagues went further to correlate antenatal placental assessment with reduced fetal movement.^25^ Noor^26^ and colleagues identified placental thickness as a promising tool for estimating fetal weight.

Kinare^27^ in India and Fang^28^ found there was significant relationship between mid pregnancy placental volume and birth weight.^27^ Campbell et al.^29^ are of the opinion that effective placental volume is a new useful parameter for identifying small for gestational age babies.

Isakov^4^ and co –workers also did their work on placental volume and observed there was a weak correlation between estimated placental volume and birth weight.^4^ We observed that only few authors like Habib^11^ carried out studies on placental diameter. She found that using PD and PT could be a good prognostic assessment for identifying retarded fetal growth.

### Limitation of the study

This is a single center study. A cohort or a wider community study would be worthwhile.

## CONCLUSION

Placental thickness and Placental diameter can be used to predict gestational age. It is therefore advised to use PT & PD in ultrasound obstetric assessment especially when last menstrual period is not clear.

### Recommendation

We recommend more studies to lay emphasis on placental diameter as most researchers concentrated more on placental thickness.

### Authors’ Contribution

**NRN & JMC,** contributed to the conception, writing and proof reading of this manuscript.

**JOO:** Contributed in data and statistical analysis of this work, while.

**NRN & JOO:** Contributed in proofreading the manuscript. All authors read and approved the final manuscript.

**JOO & NRN:** Are guarantors of the paper.

All authors contributed to the conception and writing of the manuscript.
